# Uncommon Presentation of a Rib Osteochondroma Misdiagnosed as a Breast Lesion: A Case Report

**DOI:** 10.1155/cro/8891635

**Published:** 2026-04-30

**Authors:** Bailey R. Abernathy, Christina L. Shabet, Joshua P. Castle, Maristella S. Evangelista, Michael P. Mott

**Affiliations:** ^1^ Department of Orthopedic Surgery, Henry Ford Health, Detroit, Michigan, USA, henryford.com; ^2^ Department of Surgery, Section of Plastic Surgery, University of Michigan, Ann Arbor, Michigan, USA, umich.edu; ^3^ Department of Plastic Surgery, Henry Ford Health, Detroit, Michigan, USA, henryford.com

**Keywords:** case report, chest wall mass, costal osteochondroma, rib osteochondroma

## Abstract

**Case:**

Osteochondromas are common benign bone tumors. Rarely, these lesions present on the ribs and can be concerning for a breast mass. This case discusses a healthy 21‐year‐old female with a firm, fixed, painful breast mass. Her initial ultrasound and 6‐month follow‐up ultrasound were both benign. The mass gradually increased in size with continued pain over 2 years. Almost two and a half years after presentation, the patient had an additional ultrasound and CT chest revealing a bony exostosis from the anterior aspect of the third rib. Excision was performed in a joint case with orthopedic oncology and plastic surgery, with pathology confirming the diagnosis of an osteochondroma.

**Conclusion:**

It is important to consider alternative diagnoses, such as rib osteochondroma, in the differential diagnosis of firm, fixed breast masses in young, postpubescent females.

## 1. Introduction

Osteochondromas are among the most common benign bone tumors, accounting for 20%–50% of all benign bone tumors [1, 2]. Most often, they present asymptomatically in the metaphyseal area of long bones of young males in the first 30 years of life [1, 3]. They can present as solitary lesions or as part of multiple lesions in a genetic disorder such as multiple hereditary exostosis (MHE) [3]. When present as a solitary lesion, these lesions have a propensity to arise in the metaphyseal region of long bones of the appendicular skeleton. Lesions may be sessile or pedunculated in morphology, but always are contiguous with the medullary canal and have a characteristic cartilaginous cap [1, 2].

Conservative management with continued observation is often the treatment of choice given their benign and often asymptomatic nature [1]. If there is pain associated with the lesion due to tendon, bursal or nerve irritation, decreased range of motion around a joint secondary to mechanical interference of the tumor, or for concern of malignant transformation, surgical excision is often curative. In the adolescent population, it is beneficial to postpone operative management until skeletal maturity has been achieved due to improved recurrence rates and less risk of damage to the physis [1]. Overall, with adequate resection, osteochondromas demonstrate a recurrence of less than 2% [3].

Costal osteochondromas are rare, with lesions of the ribs, pelvis, sternum, and scapula together accounting for less than 5% of these tumors [3]. Lesions are more often pedunculated rather than sessile, may present with intrathoracic or extrathoracic orientation, and most commonly arise at the costochondral junction [4]. Osteochondromas of the ribs can lead to significant complications including pneumothorax, hemothorax, diaphragmatic rupture, vascular and nerve impingement, and pleural or pericardial effusions [5, 6]. As such, surgical treatment is often preferred to prevent complications and to confirm diagnosis [1, 7].

Lesions of the chest wall, including osteochondromas, can masquerade as a breast lesion given the shared anatomic location. Adolescent breast lesions are also uncommon, but when present, are most likely benign in nature related to development of the breast tissue or benign tumors [8]. There are several documented cases of rib osteochondromas in the pediatric population [5, 7, 9, 10], and a few in postpubescent males [11, 12]; however, there is a paucity of reports of osteochondromas of the rib misdiagnosed as a benign breast mass in a postpubescent female patient [13].

## 2. Case Presentation

Written informed consent has been obtained by the patient to share this case information and digital photos for educational purposes. Institutional IRB and ethics approval were waived given that this case details a single patient. This report follows the CARE guidelines to improve the accuracy, transparency, and quality of this case report.

A 21‐year‐old female without significant past medical or family history of cancer presented with discomfort in her chest, first noted after a ball was bumped off of her chest while playing soccer. A firm, fixed, right breast mass was identified by her primary care provider in the area of her discomfort. A breast ultrasound revealed a nonsuspicious lesion deemed negative for malignancy. A repeat breast ultrasound performed 6 months later again reported no evidence of malignancy, and she was told this was consistent with dense breast tissue.

**Figure 1:**
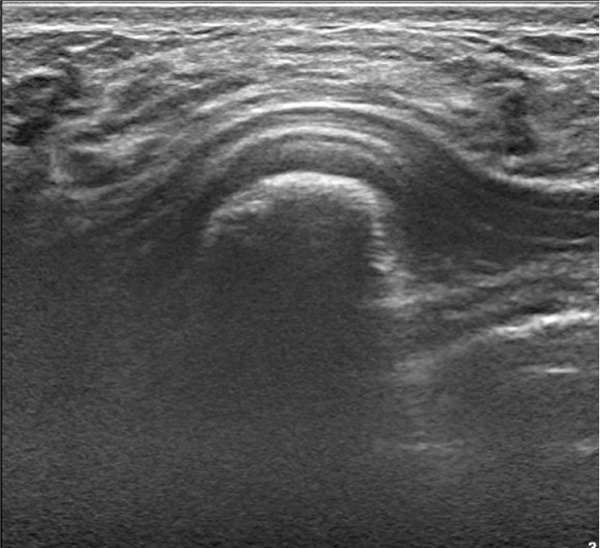
Right breast ultrasound demonstrating abnormal mass.

**Figure 2:**
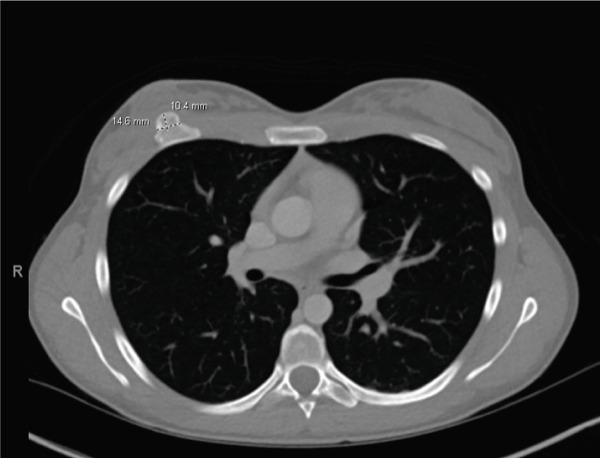
CT with IV contrast identifying bony lesion on the anterior aspect of the right third rib.

**Figure 3:**
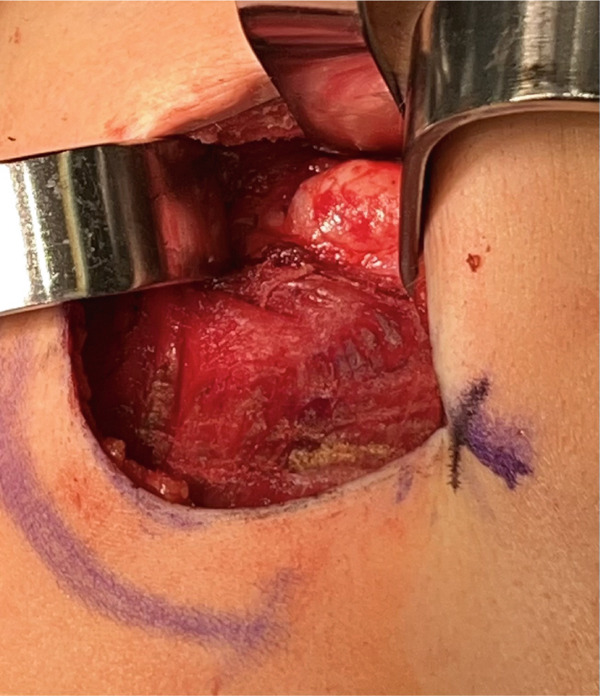
Intraoperative photo of the subpectoral approach to the third rib revealing a bony exostosis.

**Figure 4:**
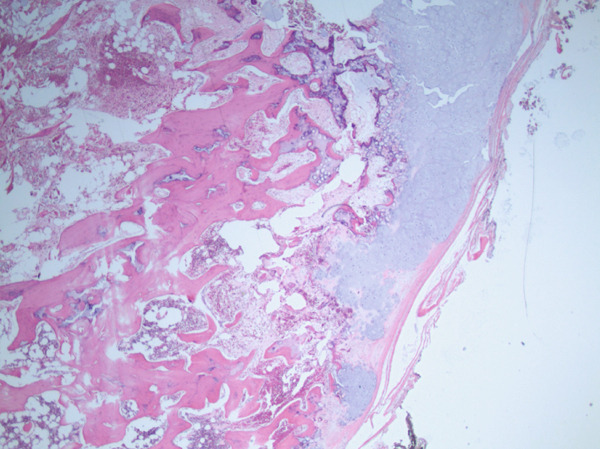
H&E stain, low power view of osteochondroma (20×) demonstrating cartilage cap lined by perichondrium that is contiguous with mature bone.

**Figure 5:**
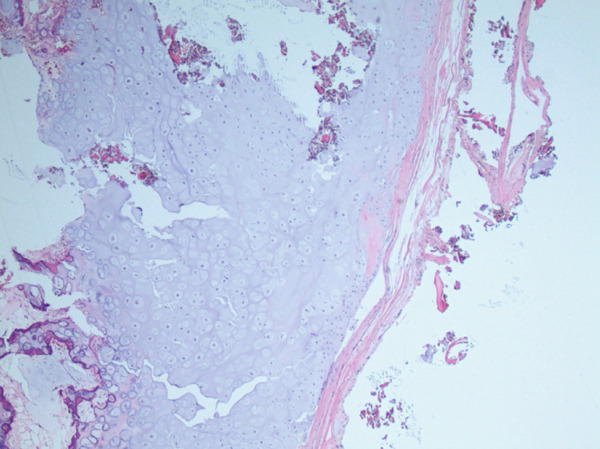
H&E stain, higher power view of osteochondroma (40×) demonstrating cartilage cap with endochondral ossification and no cellular atypia.

Over the next 2 years, the patient continued to have intermittent, sharp, shooting pain over the area of the lesion and noticed a slow increase in the size of the lesion with no fluctuations or mechanical symptoms. On exam, the lesion was noted to be 3 cm from the nipple in the eight o′clock position. A third breast ultrasound was performed 2.5 years after initial presentation. This study revealed a subpectoral mass potentially arising from the rib and the reading radiologist recommended a CT scan for further characterization of the mass (Figure 1). A chest CT scan demonstrated a bony exostosis arising from the anterior third rib, concerning for an osteochondroma, with a cartilaginous cap of 9.1 mm (Figure 2). The patient was referred to an orthopedic oncologist the following month at which time she had the first x‐ray of her ribs that again showed an osseous exostosis of the anterior right third rib. She was scheduled for complete excision of exostosis by orthopedic oncology in conjunction with plastic surgery given the location of the lesion (Figure 3). During this procedure, an inframammary approach was used to dissect to the pectoralis fascia and utilize a subfascial plane to visualize the mass. The mass was removed en bloc using an osteotome. The pleura was not breached, and no chest tube was required; however, a Blake drain was placed and removed postoperative Day 3. Pathology identified the lesion as an osteocartilaginous lesion comprised of a hyaline cartilaginous cap with a mild increase in cellularity but no significant cytologic atypia, confirming the diagnosis of an osteochondroma (Figures 4 and 5). Postoperative radiographs showed no pneumothorax, rib fractures, or evidence of early recurrence. At 1‐year follow‐up, the patient was doing well with resolution of chest discomfort and no evidence of recurrence of the mass seen on a screening chest CT.

## 3. Discussion

Osteochondromas originating from the ribs are exceedingly rare. The present case report highlights the importance of considering alternative diagnoses, such as a rib osteochondroma, when evaluating breast lesions or masses. In this case, the misdiagnosis resulted in a significant delay in diagnosis and management. The presentation, imaging findings, and surgical management of this third rib osteochondroma highlight a unique situation for a postpubescent female and contribute to the limited existing literature on delayed diagnosis of rib osteochondromas. Osteochondroma should be considered in the differential diagnosis in a young adult presenting with a firm, fixed breast mass and appropriate imaging studies, including plain radiographs even in the setting of a negative ultrasound, should be obtained to prevent late identification of an osseous process.

A thorough workup should be completed for all tumors that affect the ribs, as only 10% are benign [10]. Diagnosis of an osteochondroma of the rib typically begins with a chest radiograph to visualize the lesion, followed by advanced imaging in the form of a CT scan or MRI to identify the intramedullary continuity of the lesion [6]. This imaging also allows for assessment of the size of the cartilage cap, which serves as the best indicator for malignancy [1, 6, 9]. A bone scan may demonstrate increased scintigraphic uptake if a lesion is undergoing malignant transformation [14]. In the pediatric population, where many of these lesions present, an ultrasound can also be helpful while limiting radiation exposure [14–16]. In the author′s experience, we prefer to initially obtain radiographs followed by CT scans to work up these bony lesions involving the rib cage. Consideration for obtaining additional advanced imaging such as MRI or bone scans may be considered if there are lytic features, a large cartilage cap, or soft tissue extension of the mass. In this case, the CT was diagnostic following a negative ultrasound study given the anchoring bias of the ultrasound focusing on breast tissue as opposed to a CT to evaluate the chest wall.

A review of the limited number of case reports of costal osteochondromas demonstrates the rarity of the diagnosis and the various treatment options. Bakshi et al. reported on a case series of seven osteochondromas of the chest wall treated surgically due to the following indications: pain, confirmation of diagnosis, recurrent pneumothorax, and malignant transformation [7]. Tiwari et al. and Marino‐Nieto et al. described two 7‐year‐old patients with asymptomatic solitary lesions treated with excision to prevent complications [5, 10]. Morales et al. presented the case of a 14‐year‐old who suffered a diaphragmatic laceration from an osteochondroma leading to hemopneumothorax prior to excision [6]. These cases differ from the one presented here as these patients were diagnosed as prepubescent children as opposed to our patient with onset of symptoms in early adulthood. One case details a male patient diagnosed at 23 years of age; however, given that our patient is female with developed breast tissue, her diagnosis was comparatively delayed [4].

Surgical treatment of costal osteochondromas is not always necessary depending on the clinical features. Kadu et al. reported on a case of a child who was successfully treated nonoperatively for an asymptomatic costal osteochondroma with serial radiographs through their adolescent years [9]. Although not all lesions require surgical management, it is essential to thoroughly counsel patients about their diagnosis, understand their symptom burden, and discuss the potential risks of observing versus surgical resection. In the case presented here, given that the patient was experiencing pain and it was interfering with her hobbies and daily life, surgical excision was the elected treatment.

The present case contributes to the current literature detailing a unique case of a postpubescent female with a delay in care of a rib osteochondroma due to a misdiagnosis of a breast mass. This case is limited in its generalizability given that it is a single case and was complicated by a delay in diagnosis. Although rare, this case highlights the importance of considering a rib osteochondroma when evaluating breast masses that are firm and immobile.

## Funding

No funding was received for this manuscript.

## Consent

The patient allowed personal data processing and informed consent was obtained from all individual participants included in the study.

## Conflicts of Interest

The authors declare no conflicts of interest.

## Data Availability

Data sharing is not applicable to this article as no datasets were generated or analyzed during the current study.
